# Prevalence and influencing factors of dry eye syndrome among pilots: A survey study

**DOI:** 10.1371/journal.pone.0344244

**Published:** 2026-03-17

**Authors:** Mingyue Zhang, Tiebing Liu, Yanchuang Liang, Yanmin Qi, Xin Li, Qingjun Hu

**Affiliations:** 1 Ophthalmology Department, Civil Aviation General Hospital, Beijing, China; 2 Civil Aviation Public Health Emergency Management Office, Civil Aviation Medicine Center, Civil Aviation Administration of China (Civil Aviation General Hospital), Beijing, China; 3 Office of Aviation Health, Civil Aviation Administration of China (Civil Aviation General Hospital), Beijing, China; 4 Civil Aviation Medical Assessment Institute, Civil Aviation Administration of China (Civil Aviation General Hospital), Beijing, China; Saarland University, GERMANY

## Abstract

**Background:**

Dry eye syndrome (DES) is a prevalent ocular condition that significantly impacts affected individuals’ quality of life and occupational performance. This study investigates the prevalence and contributing factors of DES among pilots, which is a group particularly susceptible to environmental and occupational stressors.

**Methods:**

A descriptive, observational study was conducted, which involved 794 pilots. Based on the severity of DES, these pilots were assigned into mild, moderate and severe groups. Data was collected through surveys, and analyzed using multiple linear regression, in order to determine the relationship between the DES scores and potential influencing factors.

**Results:**

The study revealed that all pilots included in the present study were affected by DES, in which 88.40% of pilots experienced moderate DES and 11.60% of pilots reported severe DES. After adjusting for other covariates in the model, the multivariate analysis revealed that eyelid diseases, ocular surface disease, poor sleep quality, and fatigue were statistically significant and positively correlated to higher DES scores (*p* < 0.05), while residing in the southern region and engaging in physical activities were statistically significant and negatively correlated to the DES scores (*p* < 0.05).

**Conclusion:**

The high prevalence of DES in pilots highlights the urgent need for tailored occupational health interventions. Strategies to mitigate DES risk should include promoting regular physical exercise, improving sleep quality, and addressing fatigue. Future research should prioritize longitudinal studies to establish causal relationships and develop targeted management approaches for this high-risk occupational group.

## Introduction

Dry eye syndrome (DES) represents as one of the most prevalent ocular disorders, and is the leading cause of clinical presentations related to eye discomfort [1]. Globally, the prevalence of DES is estimated to range from 5% to 50% [2], and higher prevalence have been observed in the elderly and individuals with specific systemic or ocular conditions [3]. DES is a multifactorial ocular surface disease characterized by tear film instability, hyperosmolarity, ocular surface inflammation, and neurosensory dysfunction [4]. These pathophysiological changes manifest as symptoms, such as dryness, grittiness, burning sensations, foreign body sensation, and visual disturbances [2]. DES significantly impacts a patient’s quality of life, affecting daily activities and overall well-being.

The aviation industry poses distinct risks for ocular health, with pilots exposed to high-altitude, hypobaric/low-pressure conditions, and oxygen mask use (in military aviation). Additional factors, such as prolonged exposure to low-humidity cabin environments and sustained visual focus during flights, may further exacerbate tear film instability and ocular surface damage, although these are also relevant to other settings (*e.g.,* computer use). Despite these challenges, research that focuses on DES in pilots remain limited. To date, evidence remains strikingly sparse, and merely a single study has been published, which reports a 72.30% prevalence for DES in Australian pilots [5]. Furthermore, previous studies have highlighted the high prevalence of DES in individuals with extensive screen time and exposure to air-conditioned environments [6]. Addressing DES among pilots is important not only to safeguard their ocular health and well-being, but also to mitigate potential occupational challenges. DES-induced discomfort, visual disturbances, and fatigue may impair a pilot’s ability to maintain optimal concentration and visual acuity during flight operations [7]. Without timely intervention, the progression of DES in this occupational group can lead to diminished performance, posing potential risks to flight safety [8].

Understanding the underlying factors that influence DES in pilots is crucial for designing targeted and effective interventions to prevent and manage this condition. The present study aims to address a gap in research by investigating the prevalence of DES in pilots, and exploring factors associated to its severity. Using a cross-sectional design, the study focused on identifying modifiable factors that may inform potential occupational health strategies. Although the present study cannot establish causality, it seeks to provide foundational evidence that can support the future development of tailored health policies and preventative measures for pilots.

## Materials and methods

### Subjects

**Fig 1:**
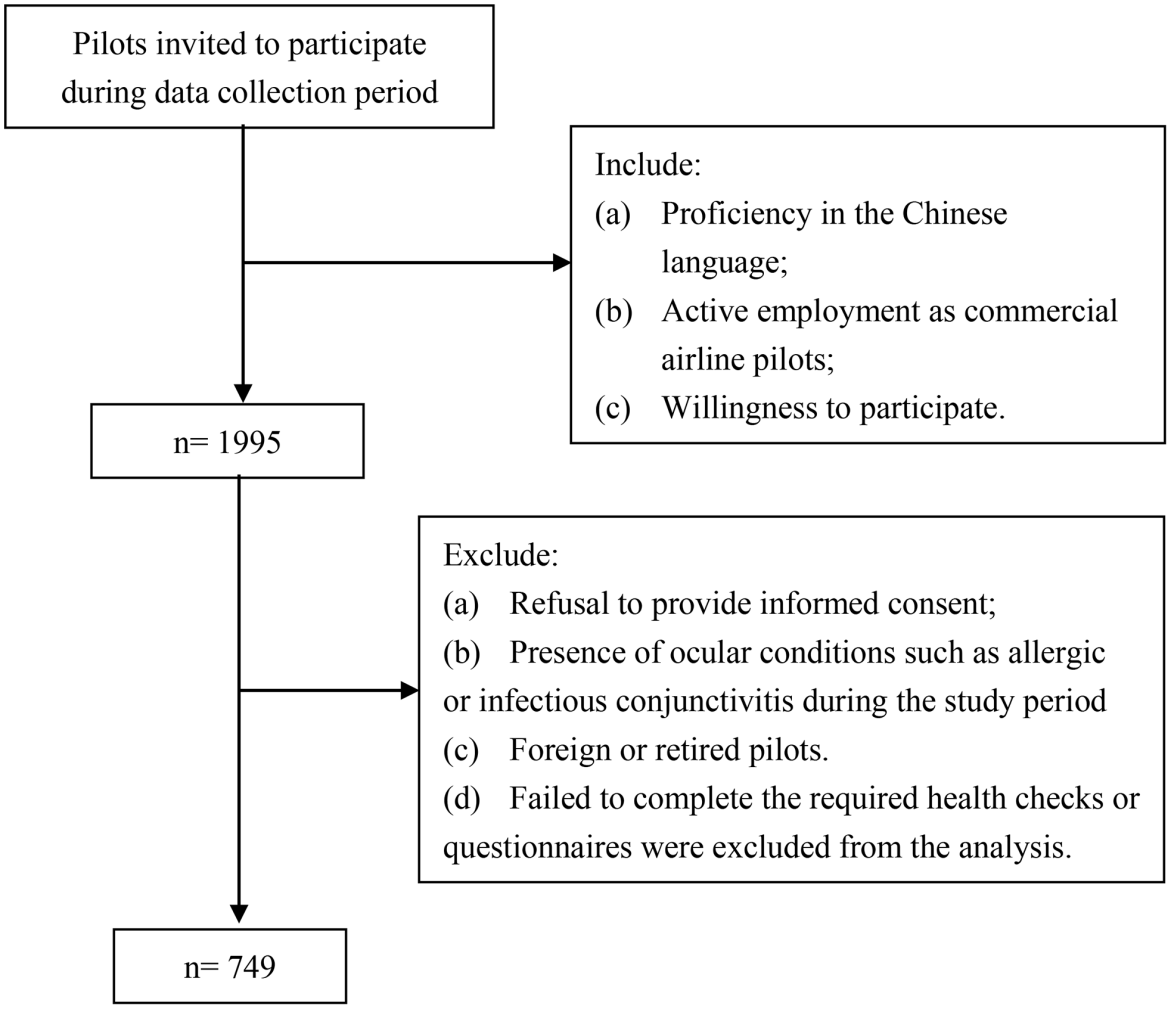
Flowchart for the participant recruitment of the study on dry eye syndrome in pilots.

The present cross-sectional study included a purposive sample of 794 pilots recruited from six Chinese airlines during the period of January to March 2024 (Fig 1). The study protocol was reviewed and approved by the Ethics Committee of Civil Aviation General Hospital (No. 2023-L-K-42), and performed in accordance with the ethical standards laid out in the 1964 Declaration of Helsinki. Informed consent was obtained from all participants. The consent process was implemented through a mandatory item in the questionnaire, which stated: “Do you agree that your anonymized responses will be used for data analysis in this research project and be published?” Merely participants who selected “Yes” were able to proceed with and complete the survey. This method provided a digital record of consent for each participant. No minors were included in the study. The participant inclusion criteria included the following: (a) proficiency in the Chinese language, (b) active employment as commercial airline pilots, and (c) willingness to participate. The participant exclusion criteria included the following: refusal to provide an informed consent, the presence of ocular conditions such as allergic or infectious conjunctivitis during the study period, and foreign or retired pilots. In addition, pilots who failed to complete the required health checks or questionnaires were excluded from the analysis. The questionnaire was electronically distributed, ensuring the participants’ ease of access and completion across various locations. All questionnaires were inspected by trained researchers.

### Demographic questionnaire

The questionnaire captured the demographic (age, education, height, weight, smoking, alcohol use, physical activity, eyeglass use, and sleep, fatigue), occupational (years of service, position, aircraft type, and flight duration), and medical data. Systemic conditions (hypertension, dyslipidemia, diabetes, thyroid, and rheumatologic disease), ocular disorders (eyelid abnormalities and other eye diseases), and ophthalmic history (long-term use of preservative-containing eye drops, prior corneal refractive surgery, and contact lens wear) were documented.

### Ocular Surface Disease Index (OSDI) questionnaire

The OSDI questionnaire was utilized to assess the prevalence and severity of DES symptoms [9]. The severity of DES was exclusively defined and categorized based on the OSDI score. The 12 components of the OSDI survey were rated on a scale from 0 to 4, where 0 indicated ‘none of the time’, 1 indicated ‘some of the time’, 2 indicated ‘half of the time’, 3 indicated ‘most of the time’, and 4 indicated ‘all the time’. The overall OSDI score was calculated using the following formula: OSDI = [(total score for all questions answered) × 100] / [(total number of questions answered) × 4]. The resulting score ranged from 0 to 100, with higher scores indicating greater symptom severity and disability. Based on the OSDI score, the severity of DES was classified into mild (13–22 points), moderate (23–32 points), or severe (33–100 points).

### Statistical analysis

The mean value, frequency and percentages for the general information, health behaviors, and other variables were calculated. In comparing subgroups, with respect to the OSDI scores, Student’s *t*-test and analysis of variance (ANOVA) were used when the dependent variables were normally distributed, and had variance homogeneity in the subgroup. Otherwise, Mann-Whitney *U*-test and Kruskal-Wallis *H*-test were used. Meanwhile, bivariate linear regression was used to reveal the correlation between OSDI scores and potential influencing factors. A multiple linear regression analysis model was used to control potential confounding factors. All variables that were identified to be significant in the bivariate analysis, and all those considered to be of interest for the study were included in the model. Prior to the regression analysis, assumptions of normality, homoscedasticity, and multicollinearity were tested to ensure the validity of the regression model. The normality of residuals was checked using graphical methods, such as Q-Q plots, and homoscedasticity was assessed through visual inspection of the residual plots. Collinearity diagnostics were performed using variance inflation factors (VIFs). The statistical analysis was conducted using the R software (version 4.3.3). The statistical tests were two-sided, and *p* < 0.05 was considered statistically significant.

## Results

### Characteristics of the study population

The socio-demographic and clinical characteristics of the participants are summarized in Table 1. Among the 1,000 pilots invited to participate in the present study, 883 pilots consented to participate, yielding a response rate of 88.30%. After screening, the data obtained from 794 pilots were deemed eligible for analysis. All subjects were male, and the mean age was 34.30 years old (standard deviation [SD]: 8.00 years old, median: 33 years old, interquartile range [IQR]: 39–28 years old, range: 23–63 years old). The mean body mass index (BMI) was 24.10 kg/m^2^ (SD: 2.30 kg/m^2^). The median duration of service was 10 years (IQR: 5–15 years). Among the 794 pilots, 36.80% of these pilots were from the northern region, and 63.20% of these pilots were from the southern region. Captains accounted for 45.60% of the cohort, while 54.40% of the cohort were co-pilots. Majority of the pilots (73.8%) operated short-haul routes, while the remaining 26.20% operated medium- to long-haul routes. For the health conditions, 3.70% of pilots reported a diagnosis of hypertension, 14.00% had dyslipidemia, 8.70% had eyelid disorders, 7.30% had other dry eye-related diseases (glaucoma and chronic conjunctivitis), and 11.10% had immune disorders. Furthermore, over half (51.70%) of the participants were classified as overweight or obese, 30.20% of the participants were smokers, 69.40% of the participants consumed alcohol, and 94.70% of the participants engaged in physical activities. The median OSDI score, which is indicative of DES severity, was 27.00 (IQR: 25.00–35.00).

**Table 1:** Sociodemographic and clinical characteristics of participants (*N* = 794).

Characteristics	Values
Age, years, median (range)	33 (23-63)
BMI, kg/m^2^, mean (SD)	24.10 (2.27)
Overweight/Obesity, *n* (%)	381 (51.70%)
Physical activity, *n* (%)	
<1 time/week	45 (5.67%)
1-2 times/week	374 (47.10%)
≥3 times/week	375 (47.20%)
Smoking, *n* (%)	240 (30.20%)
Alcohol drinking, *n* (%)	551 (69.40%)
Hypertension, *n* (%)	29 (3.65%)
Dyslipidemia, *n* (%)	111 (14.00%)
Eyelid diseases, *n* (%)	69 (8.69%)
Eye diseases, *n* (%)	58 (7.30%)
Wearing glasses, *n* (%)	234 (29.50%)
Length of service, years, median (IQR)	10.00 (5.00, 15.00)
Position, *n* (%)	
Captain	362 (45.60%)
Co-pilot	432 (54.40%)
Aircraft type, *n* (%)	
Narrow-body aircraft	721 (90.80%)
Wide-body aircraft	73 (9.19%)
Flight Duration, *n* (%)	
Short-haul routes (≤3 hours)	586 (73.80%)
Medium to long-haul routes (>3 hours)	208 (26.20%)
Sleep score, *n* (%)	
0-5 points	439 (56.60%)
6-10 points	271 (34.90%)
11-15 points	59 (7.60%)
16-21 points	7 (0.90%)
Fatigue, *n* (%)	305 (38.40%)
Dry eye syndrome score, median (IQR)	27.00 (25.00, 35.00)

Notes: BMI, body mass index; SD, standard deviation; IQR, interquartile range.

### OSDI questionnaire

**Fig 2:**
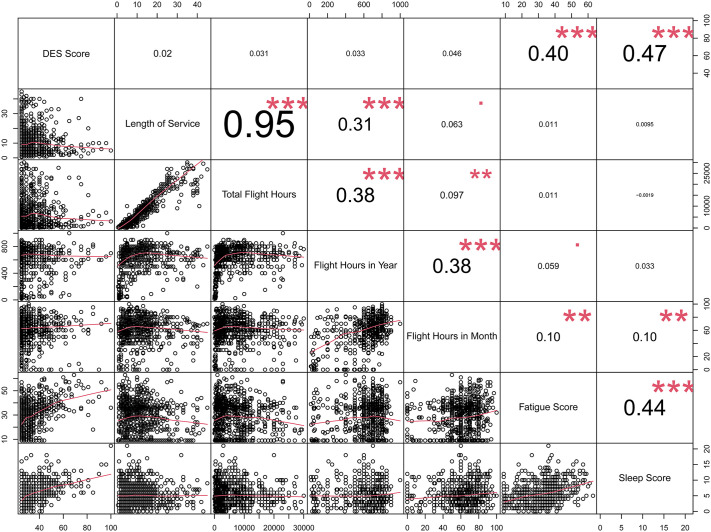
Plots for the correlation of dry eye Ocular Surface Disease Index (OSDI) scores with different variables. The array plot illustrates the correlation between dry eye OSDI scores and selected variables. Each cell displays the correlation coefficient, indicating the strength and direction of the association. The significance levels were denoted by asterisks: ^*^p < 0.05, ^**^p < 0.01, and ^***^p < 0.001. The plot provides a comprehensive comparison of the relationships across variables.

Remarkably, all participants in the present study were diagnosed with DES, underscoring its universal prevalence within this cohort of pilots. The severity of DES was further classified based on the OSDI scores. Among the 794 participants, the majority (701 individuals, accounting for 88.30%) were categorized as having moderate DES. Meanwhile, the remaining 93 participants (11.70%) were classified as suffering from severe DES. The correlation analysis between the dry eye OSDI scores and various variables is presented in the array plot (Fig 2). Significant correlations were observed across multiple variables, which included OSDI score *vs.* fatigue score and sleep score, hours in month *vs.* fatigue score and sleep score, and fatigue score *vs.* sleep score, respectively. Notably, variables that included the fatigue score and sleep score suggested a strong and consistent association with the OSDI scores. Furthermore, variables that included hours in month *vs.* fatigue score and sleep score indicated significant, though comparatively weaker relationships.

### Bivariate associations between independent variables and quality of life

The DES score, which was derived from the OSDI questionnaire, was utilized as a continuous dependent variable in the linear regression analysis. The analysis of the relationship between the sociodemographic and psychosocial characteristics of the participants, and DES scores are presented in Table 2. The univariate linear regression results revealed that flying a wide-body aircraft, having dyslipidemia, eyelid diseases, or ocular surface disease, wearing glasses, poor sleep quality, and fatigue were positively linked to higher DES scores. Conversely, residing in the southern region and engaging in physical activities were negatively associated to the DES scores.

**Table 2:** Univariate and multiple linear regression analyses of factors associated to DES in pilots.

Variable	Univariate linear regression analysis		Multiple linear regression analysis	
	95% CI	*p*-value	95% CI	*p*-value
**Region**				
North	0.00 (Reference)		0.00 (Reference)	
South	−5.59 (−7.22, −3.95)	<0.001	−4.63 (−6.25, −3.01)	<0.001
**Age**	−0.04 (−0.14, 0.06)	0.471		
**Year of service**	−0.03 (−0.12, 0.07)	0.576		
**Flight duration**				
Short-haul routes (≤3 hours)	0.00 (Reference)			
Medium- to long-haul routes (>3 hours)	0.97 (−0.87, 2.81)	0.303		
**Aircraft type**				
Narrow-body aircraft	0.00 (Reference)			
Wide-body aircraft	3.39 (0.59, 6.18)	0.018		
**Flight year**	−0.03 (−0.13, 0.06)	0.507		
**Total hour**	−0.00 (−0.00, 0.00)	0.757		
**Year hour**	0.00 (−0.00, 0.00)	0.936		
**Month hour**	0.02 (−0.02, 0.06)	0.389		
**Cross-time zone**	2.25 (−0.10, 4.60)	0.060	−0.71 (−3.38, 1.95)	0.599
**Physical activity, *n* (%)**				
<1 time/week	0.00 (Reference)		0.00 (Reference)	
1-2 times/week	−5.21 (−8.80, −1.63)	0.004	−3.38 (−6.61, −0.16)	0.039
≥3 times/week	−4.55 (−8.14, −0.97)	0.013	−3.03 (−6.26, 0.20)	0.066
**Dyslipidemia**	2.76 (0.43, 5.09)	0.020		
**Eyelid disorders**	8.46 (5.64, 11.27)	<0.001	4.85 (2.23, 7.47)	<0.001
**Ocular surface disease**	8.65 (5.60, 11.71)	<0.001	4.07 (1.31, 6.84)	0.004
**Wearing glasses**	1.82 (0.05, 3.59)	0.044		
**Sleep quality**				
Very good sleep quality.	0.00 (Reference)		0.00 (Reference)	
Fair sleep quality.	8.05 (6.43, 9.66)	<0.001	6.71 (5.15, 8.27)	<0.001
Poor sleep quality	13.81 (10.91, 16.71)	<0.001	11.38 (8.60, 14.16)	<0.001
Very poor sleep quality	19.01 (11.04, 26.97)	<0.001	14.51 (6.95, 22.08)	<0.001
**Fatigue**	6.89 (5.30, 8.49)	<0.001	3.89 (2.37, 5.41)	<0.001
**Dry eye syndrome score**	−0.88 (−2.56, 0.79)	0.301		

Note: Merely statistically significant variables are presented. DES, dry eye syndrome; 95% CI, 95% confidence interval

### Multivariate linear regression

The multivariate linear regression results are presented in Table 2. After adjusting for other covariates, which included age, BMI, obesity, physical activity, smoking, drinking, wearing glasses, and position in the model, the multivariate analysis revealed that eyelid diseases, ocular surface disease, poor sleep quality, and fatigue were statistically significant (*p* < 0.05) and positively correlated to the DES score, while living in the southern region and engaging in physical activities had a statistically significant and negative correlation with the DES score (*p* < 0.05).

## Discussion

DES exhibits a global prevalence that ranges from 5% to 34%, with notably higher rates observed in Asian populations. In the field of aviation medicine, although DES has been relatively well-studied in cabin crews, research that focuses on commercial pilots remain limited. According to the International Civil Aviation Organization (ICAO) airworthiness standards, the humidity in both cabin and cockpit environments is maintained at approximately 15%. During cruising altitude, cabin pressure is typically regulated to an equivalent of 8,000 ft (~2,400 m), corresponding to an atmospheric pressure of 52.40–54.50 kPa. Commercial aviators, particularly pilots, are chronically exposed to multiple risk factors for DES, including hypoxic conditions, low humidity, prolonged use of contact lenses, extended and nighttime flight durations, and the use of preservative-containing eye drops. Pilots face additional occupational hazards, such as prolonged exposure to suboptimal lighting conditions (excessive glare or insufficient illumination), and sustained focus on instrument panels or screens, which may contribute to visual fatigue and exacerbate DES.

Existing studies have suggested that high-altitude hypoxia and hypobaric environments can activate immune responses, leading to the release of inflammatory cytokines that further damage the ocular surface microenvironment. In the present study, all participating pilots scored ≥23 on the OSDI, indicating moderate-to-severe DES, which aligns with the findings reported by prior research.

The present study assessed the association between the DES scores, and behavioral and occupational factors through the Chinese version of the OSDI questionnaire using a moderately large sample. The present study exclusively focused on male pilots due to the limited availability of female pilots in China’s civil aviation industry. Overall, after adjusting for covariates, including age, BMI, obesity, physical activity, smoking, drinking, eyeglass use, and position, the multivariate analysis revealed that eyelid diseases, ocular surface disorders, poor sleep quality, and fatigue were positively associated to the DES scores. In contrast, living in southern China (where humidity levels are generally higher than in the drier northern region) and regular physical activity were negatively associated to the DES scores.

For pilots, the relationship between DES, and fatigue or sleep disturbances remain poorly explored, in which merely one prior study focused on DES in this population. In the present study, it was identified that poor sleep quality and fatigue were positively associated to the DES score, which is similar to the findings of several previous studies [10, 11]. In the study conducted by Magno [10], after correcting for all comorbidities, dry eyes were identified to be associated to poor sleep. In the study conducted by Yu [11] on a large, community-wide scale, it was revealed that there was a significant correlation between poor sleep quality and the exacerbation of DES. Poor sleep quality and fatigue have been increasingly recognized as contributing factors to the pathogenesis of DES [10]. These conditions are associated to systemic inflammation, which is characterized by elevated levels of pro-inflammatory cytokines, such as interleukin-6 (IL-6), interleukin-1β (IL-1β), and tumor necrosis factor-alpha (TNF-α), exacerbating ocular surface inflammation and tear film instability [12]. In addition, sleep deprivation disrupts the hypothalamic-pituitary-adrenal (HPA) axis, leading to cortisol dysregulation, which in turn, impairs tear production and ocular surface homeostasis [13]. Furthermore, fatigue and poor sleep are linked to increased oxidative stress and autonomic nervous system imbalance. Both of which negatively affect lacrimal gland function and tear composition, further contributing to the hyperosmolarity characteristic of DES [14]. Moreover, poor sleep quality may heighten central pain sensitivity, intensifying subjective symptoms, such as burning and grittiness, even in cases with mild objective ocular surface findings [15]. Behavioral factors, such as increased screen time and reduced blink rates associated to sleep deprivation, further exacerbate DES symptoms [16]. These findings highlight the necessity of addressing sleep disturbances and fatigue as part of the comprehensive approach to DES management. These studies imply that addressing either of these issues can potentially ease the symptoms of the other.

The present study revealed an inverse relationship between physical activity and DES, suggesting that individuals who engage in regular physical exercise may experience fewer symptoms of DES. This finding aligns with a recent review [17] that highlighted the potential of physical activity to modulate tear film function and alleviate dry-eye symptoms through mechanisms, such as improved lacrimal gland function, reduced systemic inflammation, and enhanced autonomic balance. Physical activity has been shown to reduce oxidative stress and inflammatory markers, including IL-6 and C-reactive protein (CRP), which are associated to the pathogenesis of DES [18]. In addition, exercise may improve endothelial function and microcirculation, potentially enhancing ocular surface perfusion and tear production [19]. Despite the heterogeneity across study populations, designs, and methodologies, the growing body of evidence supports the significant role of physical activity in promoting ocular health, and reducing the burden of DES [18]. For instance, regular moderate exercise has been associated to better subjective and objective measures of tear film quality, including improved tear break-up time (TBUT) and reduced osmolarity [20]. Furthermore, physical activity may mitigate risk factors for DES, such as systemic diseases, including diabetes and metabolic syndrome, which are linked to tear film instability [21]. By encouraging active lifestyles, healthcare providers can potentially offer low-cost and accessible interventions to reduce the prevalence and severity of DES, while contributing to overall systemic health benefits. This collective evidence underscores the significant role physical activity may play in maintaining ocular health and comfort.

### Implications for occupational health

In the present study, DES symptoms were defined as self-reported ocular discomfort (*e.g.,* dryness, irritation, or visual disturbances), with or without clinical signs, as assessed by the OSDI questionnaire. Although DES management often includes universal measures, such as artificial tears (as rightly noted by the reviewer), the present findings highlight the occupational factors that may exacerbate DES in pilots, warranting tailored considerations. For instance, the cockpit environment (low humidity and prolonged visual demands) and irregular shift patterns can contribute to symptom severity. Therefore, in addition to standard therapies (*e.g.,* lubricating drops for mild cases), aviation-specific strategies, such as optimizing in-flight humidity, scheduling breaks during long-haul flights, and facilitating access to ocular health screenings, may help mitigate DES in this high-risk group.

However, the investigators acknowledge that the present study lacks a control group. Thus, it could not be definitively concluded whether the identified risk factors (*e.g.,* flight duration) are unique to pilots. Rather, these findings suggest that occupational demands may amplify DES burden, necessitating further comparative research. Future studies should determine whether pilots with DES (particularly pilots with OSDI scores ≥14) benefit more from targeted workplace interventions *vs.* standard care alone.

The present study acknowledges several limitations that warrant consideration for interpreting the findings, and guiding future research. First, the cross-sectional design precludes the establishment of causal relationships between identified risk or protective factors, and the prevalence of DES. Longitudinal studies with larger and more diverse sample sizes are needed to explore these relationships over time, and strengthen the evidence base. Second, the accuracy of self-reported data on health behaviors may be affected by recall bias or social desirability bias, potentially leading participants to overestimate or underestimate their behaviors. This limitation has been well-documented in previous studies, and remains a challenge for survey-based research. Third, the present recruitment strategy may have introduced selection bias, since individuals who are more health-conscious or motivated to improve their well-being may have been more inclined to participate, potentially skewing the findings. In addition, in order to simplify the data collection process and maximize the response rates, the investigators did not gather detailed information on critical variables, such as time-zone crossings, shiftwork schedules, and screen time during both work and leisure hours. These factors are likely to have a significant impact on DES, and should be prioritized in future research endeavors. Despite these limitations, the present study provides valuable insights into the prevalence and risk factors of DES in pilots, highlighting actionable strategies for mitigating its impact. By addressing the identified methodological constraints in future studies, researchers can build on this foundation to deepen our understanding, and improve interventions for occupational groups at high risk of DES.

### Future research

Future research should continue to explore the specific environmental factors that contribute to the lower prevalence of DES in certain regions. Longitudinal studies would provide deeper insights into how changes in flight schedules, routes, and physical activity levels affect DES over time. In addition, research into the efficacy of various interventions, such as specific types of physical exercises or sleep management programs, can help refine preventive strategies.

## Conclusion

The present study provides a comprehensive overview of the prevalence and influencing factors of DES in pilots, highlighting the critical need for targeted occupational health interventions. By addressing both identified protective and risk factors, it is possible to develop effective strategies to mitigate the impact of DES, and improve the quality of life and occupational performance of pilots.

## Supporting information

S1 File Supplemenatry file. (XLSX)
